# JAK3 Inhibition Regulates Stemness and Thereby Controls Glioblastoma Pathogenesis

**DOI:** 10.3390/cells12212547

**Published:** 2023-10-30

**Authors:** William Smedley, Amiya Patra

**Affiliations:** 1 Peninsula Medical School, University of Plymouth, Plymouth PL6 8BU, UK; ws630@bath.ac.uk; 2 Department of Biology and Biochemistry, University of Bath, Bath BA2 7AX, UK

**Keywords:** glioblastoma, JAK, differentiation, neurosphere and tumor therapy

## Abstract

Glioblastoma multiforme (GBM) is the most deadly brain tumor, effective treatment options for which still remain elusive. The current treatment procedure of maximal resection followed by chemotherapy has proved to be grossly insufficient to prevent disease progression and death. Despite best efforts, the maximum survival post-diagnosis is a mere 1.5 years. Therefore, there is a huge unmet clinical need to find effective therapeutic procedures to prevent the pathogenesis and relapse of GBM. Small-molecule inhibitors of signaling pathways are an attractive option to prevent various types of tumors. However, no effective small-molecule inhibitors have been successful against GBM in clinical trials. Various signaling pathways are altered and an array of signaling molecules, transcription factors (TFs), and epigenetic modifying factors have been implicated in the pathogenesis of GBM. JAK-STAT pathway alteration is an important contributor to GBM pathogenesis and relapse. Many small-molecule inhibitors of JAKs, or STAT TFs, especially JAK2 and STAT3, have been assessed for their anti-tumor activity in GBM. However, no definitive success so far has been achieved. Herein, by using two small-molecule inhibitors of JAK3, we show that they are quite effective in inhibiting GBM cell proliferation and neurosphere formation, downregulating their stemness character, and inducing differentiation into neuronal origin cells. The effect of a single treatment with the drugs, both in a serum-containing differentiation medium and in a proliferation medium containing EGF and FGF, was really strong in limiting GBM cell growth, suggesting a potential therapeutic application for these JAK inhibitors in GBM therapy.

## 1. Introduction

Glioblastoma multiforme (GBM) is a deadly brain tumor affecting many people for whom treatment options at the moment are extremely limited [1]. GBM occurs when progenitor cells of astroglial origin transform due to anomalies in signaling mechanisms that are essential for their differentiation into more mature cells. The major complication in treating GBM patients lies in the heterogeneity of the origin of the tumor [2,3,4]. Alterations in many signaling molecules, transcription factors (TFs), co-factors, and epigenetic modifying molecules have been implicated in the pathogenesis of GBM [5,6,7,8,9]. The rapid progression and invasive properties of GBM make it very hard to treat GBM patients. Current protocols for the treatment of GBM patients involve the maximal surgical removal of the tumor tissue followed by chemotherapy with the alkylating agent temozolomide and radiotherapy. However, this treatment option is highly ineffective, and the survival rate of GBM patients post-treatment remains poor, at only 1.5 years. The tumor relapses very quickly and becomes resistant to temozolomide treatment, adding complications to the treatment strategy. Therefore, there is a huge need for the development of novel treatment options to prevent not only GBM pathogenesis but also its relapse following treatment [10,11,12].

The signaling molecules of Janus-associated kinase (JAK) and TFs of signal transducer and activator of transcription (STAT) families are intricately involved in the proliferation, differentiation, and function of various cells [13]. There are four JAK family kinases, JAK1, JAK2, JAK3, and TYK2, present in various cells and involved in the activation of the STAT TFs. Whereas JAK1 and JAK2 are universally present in all cells, JAK3’s distribution has been reported to be prevalently in hematopoietic cells. Accordingly, the phenotypes of *Jak1*^-/-^ and *Jak2*^-/-^ mice are more severe compared with *Jak3*^-/-^ mice, where gross hematopoietic anomalies were reported [14,15]. Cell surface receptor ligation leads to the phosphorylation of JAK proteins in the cytoplasmic tails, which subsequently leads to the recruitment of STAT proteins and their phosphorylation by JAKs. There are six STAT proteins, STAT1, STAT2, STAT3, STAT4, STAT5, and STAT6, and their cellular distribution is variable, with STAT5 being more active in hematopoietic cells and STAT3 in other cell types. JAK-STAT signaling can be activated by a host of signaling molecules such as cytokines, growth factors, inflammatory molecules, etc., which is both beneficial and harmful depending on the context [16].

Apart from its roles in cellular proliferation, differentiation, and function, JAK-STAT signaling is a prime factor in tumorigenesis. Gain-of-function mutations in JAK and STAT molecules have been reported in both hematological and non-hematological tumors [17,18]. JAK-STAT involvement in GBM pathogenesis has been reported [19,20]. Among the JAK-STAT family members, JAK2 and STAT3’s involvement in GBM has been widely studied, and their enhanced activity has been linked to the severity of GBM [21,22]. Increased JAK-STAT signaling has been reported to maintain the stemness of GBM cells by upregulating and maintaining the expression of various stem cell genes such as CD44, NESTIN, PROMININ, PAX6, etc. [23]. Thereby, it facilitates tumor relapse following surgery and chemotherapy. Therefore, the inhibition of JAK-STAT signaling in GBM remains a possible therapeutic option. As the epigenetic machinery in tumor cells is unstable and they are prone to frequent modifications, genetic intervention to downregulate JAK-STAT activity in GBM cells is not a very attractive option. Small-molecule inhibitors of JAKs or STATs are a better option to inhibit their activity in GBM and hold promise, as reported in multiple studies. Herein, we used the specific JAK3 inhibitors WHI-P131 (4-(4′-hydroxylphenyl)-amino-6,7-dimethoxyquinazoline) and PF-956980 (((3R,4R)-4-Methyl-3-(methyl(7H-pyrrolo[2,3-d]pyrimidin-4-yl)amino)piperidin-1-yl)(pyrrolidin1-yl)methanone) to evaluate their efficacy in preventing GBM cell proliferation, inducing their differentiation, and in downregulating their stemness characteristics. Our results show that both small-molecule inhibitors of JAK3 signaling are very effective in blocking GBM cell proliferation and neurosphere formation, and in inducing differentiation into neurons and other neuronal lineage cells. Interestingly, these effects were executed by downregulating the stem cell characteristics of the GBM cells. Overall, our study demonstrates the potential therapeutic value of these JAK3 inhibitors in preventing GBM pathogenesis and relapse.

## 2. Materials and Methods

### 2.1. Cell Lines

Human GBM cell lines U87 and U251 were maintained in Dulbecco’s Modified Eagle’s Medium (DMEM, Gibco, Waltham, MA, USA) supplemented with 10% fetal bovine serum (FBS), 100 units of penicillin and streptomycin, 1% non-essential amino acids, 1% HEPES, and 0.05 mM of 2-mercaptoethanol. The cells were maintained in a 37 °C incubator with 5% CO_2_ and were regularly checked for contamination. To avoid continuous prolonged culture, a new vial from the frozen stock was revitalized every six months and used for experiments. Mycoplasma contamination was regularly checked using a PCR-based detection kit (Sigma Aldrich, St Louis, MI, USA).

### 2.2. Proliferation and Differentiation Assays

Single-cell suspensions of 5000 U87 or U251 cells were cultured in 1 mL of complete DMEM in each well of a 12-well plate. Cells were cultured in the presence or absence of 50 μM of WHI-P131 or 250 μM of PF-956980 for 7 or 14 days. An equal volume of DMSO as that of the drugs was added to the control cells. Afterward, the cells were collected, and the total number was counted according to the trypan blue exclusion method to check the effects of the drugs on GBM cell proliferation. Cellular differentiation into neuronal lineage cells in JAK3-inhibitor-treated wells and control cells was recorded on day 7 of culture using a Leica IM8 microscope.

### 2.3. CFSE Staining

A total of 1 × 10^6^ U87 or U251 cells were stained with carboxyfluoresceine succinimidyl ester (CFSE) as described previously [24]. Briefly, cells were collected via trypsinization, and a single-cell suspension was prepared. The cells were washed 2 times with PBS and stained with 2 μM of CFSE in PBS for 5 min in the dark at room temperature (RT). Following staining, the cells were washed with complete DMEM, resuspended in complete DMEM, and counted. A total of 10,000 CFSE-stained U87 or U251 cells were cultured in 1 mL of complete DMEM in each well of a 12-well plate in the presence or absence of 50 μM of WHI-P131 or 250 μM of PF-956980. Cells were imaged using a Leica IM8 microscope at D-0 and at 24 h of culture to evaluate CFSE dilution.

### 2.4. Cell Death Analysis 

#### 2.4.1. Annexin V/PI Staining

A total of 1 × 10^4^ U87 or U251 cells were cultured in 1 mL of complete DMEM in each well of a 12-well plate in the presence or absence of 50 μM of WHI-P131 or 250 μM of PF-956980. At 48 h of culture, cell death was analyzed using annexin V and propidium iodide (PI) staining. The cells were first stained with annexin V following the manufacturer’s protocol (BD Biosciences, Franklin Lakes, NJ, USA), and subsequently, PI (0.5 μg/mL) was added to the cells, and they were analyzed immediately using the FACS Aria and FACS Diva software (https://www.bdbiosciences.com/ja-jp/products/software/instrument-software/bd-facsdiva-software, Beckton Dickinson, Heidelberg, Germany).

#### 2.4.2. TUNEL Assay

A total of 1 × 10^4^ U87 or U251 cells were cultured in 1 mL of complete DMEM in each well of a 12-well plate in the presence or absence of 50 μM of WHI-P131 or 250 μM of PF-956980. At 48 h of culture, cell death was analyzed via TUNEL assay using the TUNEL Assay Kit-FITC (Abcam, Cambridge, UK) following the manufacturer’s protocol. Live and dead cells were analyzed using the FACS Aria and FACS Diva software (Beckton Dickinson, Heidelberg, Germany).

### 2.5. MTT Assay

A total of 1 × 10^4^ U87 or U251 cells/well treated with or without 50 μM of WHI-P131 or 250 μM of PF-956980 were plated in a flat-bottom 96-well culture plate. For each condition, cells were plated in triplicate. The metabolic fitness of the control and drug-treated cells was assessed 48 h later using MTT reagent following the manufacturer’s instructions. Briefly, 10 μL of MTT reagent was added to 100 μL of a medium containing the cells and incubated at 37 °C for 4 h. Subsequently, the supernatant was removed, and 150 μL of DMSO was added to solubilize the formazan crystals. Afterward, the absorbance at 570 nm was measured using a plate reader (Molecular Devices, San Jose, CA, USA).

### 2.6. DNA Methylation Analysis

Genomic DNA was isolated from U87 and U251 GBM cells cultured for 24 h in the presence or absence of 50 μM of WHI-P131 following the standard protocol. The DNA concentration and quality were determined by comparing the OD 260 and 280 nm observations. An amount of 100 ng of DNA from each sample was used to determine the methylated DNA status using the MethylFlash™ Methylated DNA Quantification Kit (Epigentek, Brooklyn, NY, USA) following the manufacturer’s protocol. The methylated DNA amount was estimated at OD 450 nm using a microplate reader (Molecular Devices), and the relative quantification of methylated DNA as a percentage of total DNA was calculated following the formula 5-mC % = [(Sample OD-ME3 OD)/S]/[((ME4 OD-ME3 OD) × 2)/P] × 100%, where ME3 is the negative control, ME4 is the positive control, S is the amount of input sample DNA in ng, and P is the amount of input positive control (ME4) in ng. All DNA samples were measured in duplicate.

### 2.7. DNMT Activity Assay

Nuclear extracts (NEs) were prepared from 1 × 10^6^ U87 or U251 cells cultured for 24 h in the presence or absence of 50 μM of WHI-P131, as described previously, and the protein amount was estimated using the BCA reagent. An amount of 10 μg of nuclear protein from each sample was used to determine the total DNA methyl transferase (DNMT) activity using the EpiQuik™ DNA Methyltransferase Activity/Inhibition Assay Ultra Kit (EpiGentek, Brooklyn, NY, USA) according to the manufacturer’s instructions. The total DNMT activity was estimated at OD 450 nm using a microplate reader (Molecular Devices), and the DNMT activity was calculated following the formula DNMT activity (OD/h/mg) = (No inhibitor OD-Blank OD)/[Nuclear protein amount (μg) × incubation time (h)] × 1000. All samples were measured in duplicate.

### 2.8. Spheroid Formation Assay

A total of 5 × 10^3^ U87 or U251 cells/mL were cultured in a proliferation medium (DMEM-F12 supplemented with a neural stem cell supplement (2%) and 20 ng/mL each of human basic fibroblast growth factor (FGF) and human epidermal growth factor (EGF)) in the presence or absence of 50 μM of WHI-P131 or 250 μM of PF-956980 in each well of a 12-well plate. The effects of the drugs on neurosphere formation were analyzed on days 7 and 14 of culture. Images of the spheres were captured using a Leica IM8 microscope.

### 2.9. Immunofluorescence Microscopy

For the immunofluorescence analysis of Ki-67 and pJAK3, U87 and U251 cells were cultured in DMEM on glass coverslips in the presence or absence of 50 μM of WHI-P131 or 250 μM of PF-956980 for 24 h. Afterward, the cells were fixed in 1% formaldehyde for 10 min at room temperature (RT). The fixed cells were permeabilized with chilled acetone for 15 seconds followed by chilled methanol for 3 min. The cells were washed 3 times with PBS and incubated with a blocking solution (1% BSA and 22.52 mg/mL of glycine in PBS) for 30 min at RT. Afterward, the cells were incubated with Ki-67 (1:100 dilution) or pJAK3 (Cell Signaling Technology, Danvers, MA, USA; 1:250) in 0.1% BSA in PBS for 45 min at RT. The cells were washed 3 times in PBS and incubated with secondary anti-rabbit Alexa Flour 555 (1:250 in 0.1% BSA in PBS) for 45 min in the dark at RT. Subsequently, the cells were washed 3 times with PBS and 2 times with dH_2_O. A drop of Fluoromount-G containing DAPI was added to the cells, and they were mounted on a slide for observation.

For the immunofluorescence analysis of CD44 and NESTIN, 5 × 10^3^ U87 or U251 cells/mL/well were cultured in 12-well plates in the presence or absence of 50 μM of WHI-P131 or 250 μM of PF-956980 for 6 days. Subsequently, the cells were processed for staining following the above protocol in the 12-well plates. Secondary anti-rabbit Alexa 555 and anti-mouse Alexa 488 were used to discriminate rabbit and mouse antibodies. DAPI (1:1000) was added to the cells in the well for 5 min at RT and washed twice before adding 250 μL of PBS to the cells. All immunofluorescence samples were imaged with a Leica IM8 fluorescence microscope.

### 2.10. Reverse Transcriptase PCR

U87 or U251 cells either left untreated or treated with 50 μM of WHI-P131 or 250 μM of PF-956980 for 8 days were used to synthesize cDNA using the Miltenyi Biotec μMACS™ One-step cDNA synthesis kit (130-091-902) and protocol. Semiquantitative RT-PCR was performed to reveal the levels of expression of indicated genes (*ACTB*: for 5′-CACACTGTGCCCATCTAC-3′, rev: 5′-TCGTAGCTCTTCTCCAGG-3′; *DNMT1*: for 5′-AAAACCCAGCCAACAGAG-3′, rev: 5′-GGACTGGACAGCTTGATG-3′; *DNMT3B*: for 5′-ACGGTTCCTGGAGTGTAA-3′, rev: 5′-TCCCCTGTTTGATCGAGT-3′; and *TUBB3*: for 5′-AACAGCAGCTACTTCGTG-3′, rev: 5′-GGTCGTTCATGTTGCTCT-3′) in untreated and inhibitor-treated cells.

### 2.11. Statistical Analysis

The data are presented as means ± sd. Statistical significance was assessed using Student’s t-test for comparisons between two groups. The *p*-values, wherever applicable, were calculated based on the means of the experimental replicates.

## 3. Results

### 3.1. Strong Influence of JAK Inhibitors on Glioblastoma Cell Proliferation

**Figure 1:**
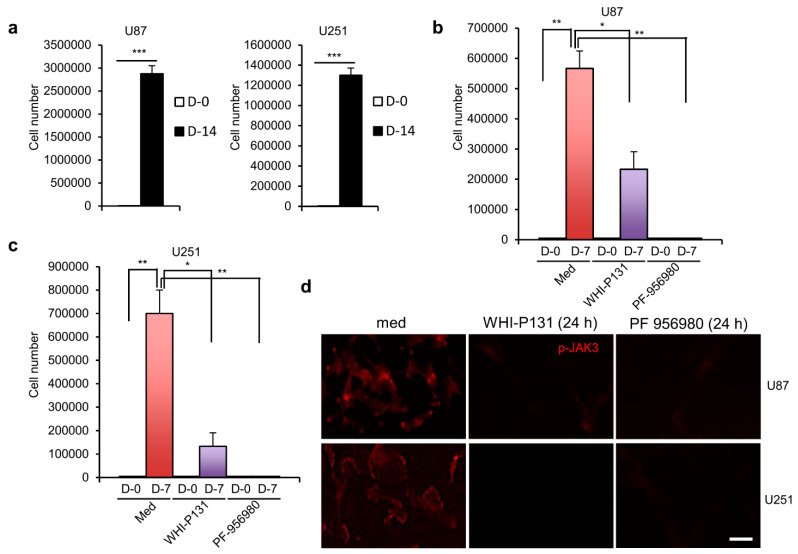
Effects of JAK3 inhibitors WHI-P131 and PF-956980 on GBM cell growth. (**a**) U87 and U251 cell numbers after 14 days of culture of 5 × 10^3^ input cells in DMEM (*** *p* < 0.0001; paired t-test). (**b**) Effect of WHI-P131 (50 μM) or PF-956980 (250 μM) on 5 × 10^3^ input U87 cell proliferation in DMEM after 7 days of culture (* *p* = 0.0377 and ** *p* = 0.0035; paired t-test). (**c**) Effect of WHI-P131 (50 μM) or PF-956980 (250 μM) on 5 × 10^3^ input U251 cell proliferation in DMEM after 7 days of culture (* *p* = 0.0234 and ** *p* = 0.0068; paired t-test). (**d**) Immunofluorescence analysis of pJAK3 levels in WHI-P131- (50 μM) or PF-956980- (250 μM) treated U87 and U251 GBM cells compared with untreated cells 24 h after culture. Scale bar: 100 μm. Data in (**a**–**c**) are presented as means ± sd and are representative of ten independent experiments. Data in (**d**) are representative of 3 independent experiments.

To check the rate of proliferation of GBM cells, we cultured U87 and U251 cells in DMEM for two weeks. Compared with the 5000 input cells at the beginning of culture, there was a 575- and 260-fold increase in the cell number after 14 days of culture for U87 and U251 cells, respectively (Figure 1a), suggesting their vigorous rate of proliferation. As the primary necessity to treat GBM patients is to inhibit the tumor cells from proliferating, we treated U87 and U251 cells with the JAK3 inhibitor WHI-P131 to investigate whether it can effectively block their extensive rate of proliferation. As shown in Figure 1b,c, WHI-P131 treatment effectively reduced the rate of proliferation for both GBM cell lines after 7 days of culture compared with the untreated control cells. We observed an even stronger effect on GBM cell proliferation with the other JAK3 inhibitor PF-956980 (Figure 1b,c). Thus, both these inhibitors efficiently blocked the proliferation of GBM cells, suggesting their potential therapeutic application against this deadly tumor. As expected, treatment with both WHI-P131 and PF-956980 blocked the activation of JAK3 in U87 and U251 cells (Figure 1d), indicating the effectiveness of these treatments.

### 3.2. JAK3 Inhibitors Specifically Block GBM Cell Proliferation

**Figure 2:**
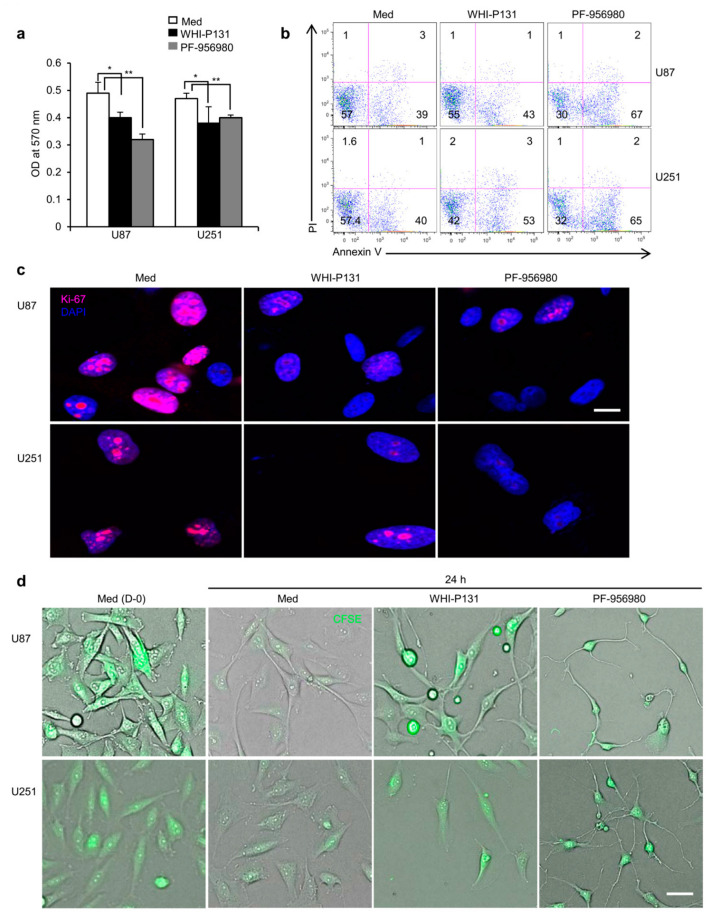
Strong anti-proliferative effects of WHI-P131 and PF-956980 on GBM cells. (**a**) Metabolic activity of WHI-P131- (50 μM) or PF-956980- (250 μM) treated U87 or U251 cells compared with respective untreated cells after 48 h of culture, as revealed by MTT assay (U87: * *p* = 0.0266 and ** *p* = 0.0034; U251: * *p* = 0.0273 and ** *p* = 0.0047; paired t-test). (**b**) Cell death analysis of U87 or U251 cells following 48 h treatment with WHI-P131 (50 μM) or PF-956980 (250 μM) compared with untreated control cells, as revealed by PI and annexin V staining. (**c**) Immunofluorescence analysis of nuclear distribution of Ki-67 molecules in untreated or JAK3-inhibitor-treated GBM cells 24 h after treatment. Scale bar: 100 μm. (**d**) Effect of WHI-P131 (50 μM) or PF-956980 (250 μM) on U87 or U251 cell cycle, as revealed by CFSE dilution assay 24 h after drug treatment. Scale bar: 100 μm. Data in (**a**–**d**) are representative of three independent experiments and in (**a**), are presented as means ± sd.

To delineate whether the strong inhibition of U87 and U251 cell proliferation in the presence of WHI-P131 and PF-956980 is a specific effect on cell proliferation or an indirect effect because of enhanced cell death, we analyzed their metabolic fitness at 48 h after treatment. As shown in Figure 2a, at 48 h, both U87 and U251 cells were equally metabolically fit as the untreated control cells. The significant increase in the OD 570 nm value for the untreated control cells in both U87 and U251 cells was not due to enhanced cell death in the JAK3-inhibitor-treated wells; rather, it was due to the increase in the cell number after 48 h in untreated wells compared with the inhibition of proliferation in the treated wells. This was clear, as annexin V and propidium iodide (PI) staining at 48 h after JAK3 inhibitor treatment did not reveal any significant increase in cell death (annexin V^+^PI^+^ cells) for either of the inhibitors in both the GBM cell lines (Figure 2b). The unaffected cell survival was further confirmed in the TUNEL assays, which showed comparable cell death in U87 and U251 GBM cells with or without WHI-P131 or PF-956980 treatment (Supplementary Figure S1). Further evaluation of Ki-67, the nuclear antigen marker for active cell proliferation, revealed its strongly diminished presence in the WHI-P131- and PF-956980-treated GBM cells compared with the untreated control cells 24 h after culture (Figure 2c). This is in line with the strongly inhibited proliferation in both these cell lines shown in Figure 1b,c. Corroborating the Ki-67 observation, the cell cycle in WHI-P131- and PF-956980-treated GBM cells was inhibited as they failed to dilute the cell-membrane-binding dye CFSE 24 h after treatment compared with the untreated control cells (Figure 2d). Overall, these observations suggest that the JAK3 inhibitors WHI-P131 and PF-956980 do not induce cell death at the given concentrations; rather, they effectively inhibit GBM cell proliferation. However, in multiple experiments, compared with a single treatment with these inhibitors, we did not observe any additive effect on cell survival or proliferation in combination treatment with both WHI-P131 and PF-956980.

### 3.3. JAK3 Inhibition Induces Differentiation of GBM Cells

**Figure 3:**
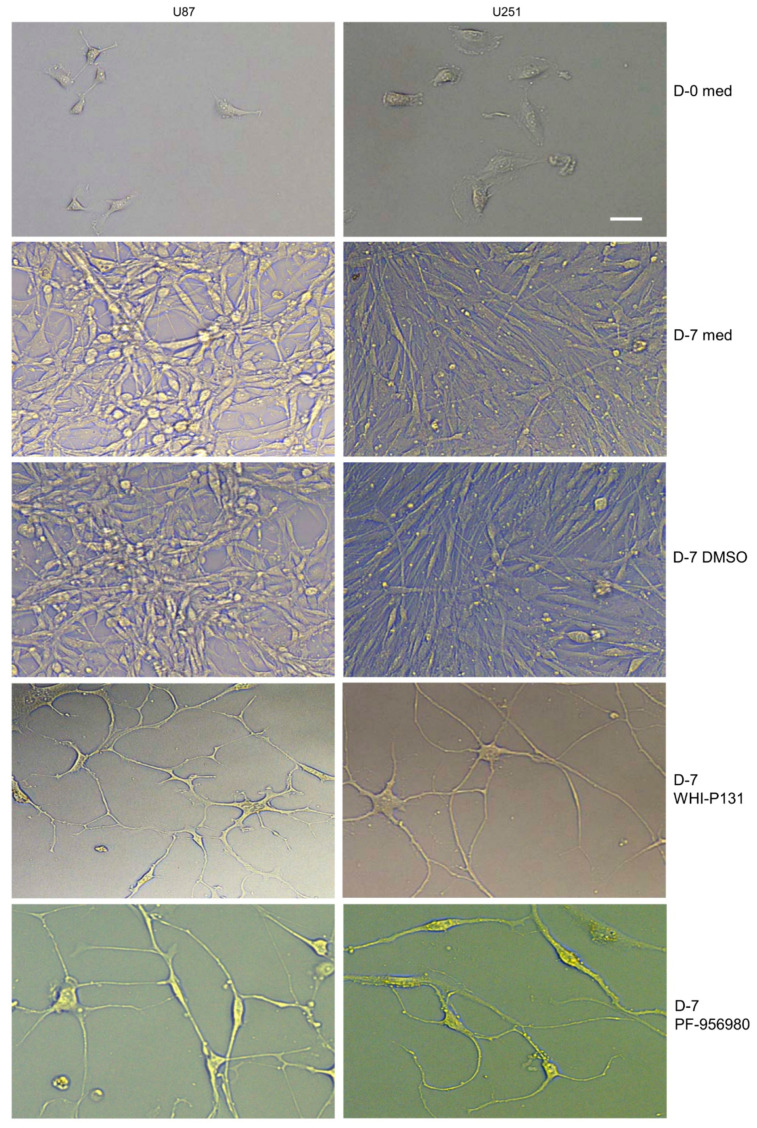
WHI-P131 and PF-956980 induce differentiation of GBM cells into neuronal lineage cells. Status of U87 and U251 cell proliferation or differentiation at day seven of culture with or without treatment with JAK3 inhibitors WHI-P131 (50 μM) or PF-956980 (250 μM). Treatment with DMSO was used as a control. Data are representative of ten independent experiments. Scale bar: 100 μm.

The lack of enhanced cell death in U87 and U251 cells in response to the WHI-P131 and PF-956980 treatments was intriguing. To check what could be the effect of inhibited proliferation on JAK3-inhibitor-treated cells, we observed them regularly. Interestingly, both the U87 and U251 cells started to differentiate, which was evident as early as 24 h after treatment (Supplementary Figure S2). By day 7 of drug treatment, both the cell lines differentiated into what mostly appeared to be fully developed neurons with extensive neural networks and connections (Figure 3). There were also phenotypically different cells of neuronal origin such as astrocytes in the treated wells, suggesting that the JAK3 inhibitors not only prevented tumor cell proliferation but also induced their differentiation into a more mature stage, in which they were not proliferating. In contrast, untreated or DMSO-treated cells proliferated extensively by day 7 and became overconfluent. The differentiation of GBM cells from highly proliferating blasts to morphologically differentiated non-proliferating neurons induced by the JAK3 inhibitors suggests that the inhibition of JAK3 activity could, in principle, be applied as a therapeutic strategy to prevent GBM. The observation that both WHI-P131 and PF-956980 induced GBM cell differentiation following similar kinetics suggests that the effect is specific to JAK3 inhibition. Most importantly, the inhibitor-treated cells remained in a differentiated and non-proliferating state until D-75 of culture, the latest time point of our observations. However, combination treatment with both WHI-P131 and PF-956980 did not bring any additional benefit to the kinetics or extent of differentiation in any of the GBM cells compared to the effects observed with a single treatment with the inhibitors.

### 3.4. Inhibition of JAK3 Strongly Reduced the Ability of GBM Cells to Form Neurospheres

**Figure 4:**
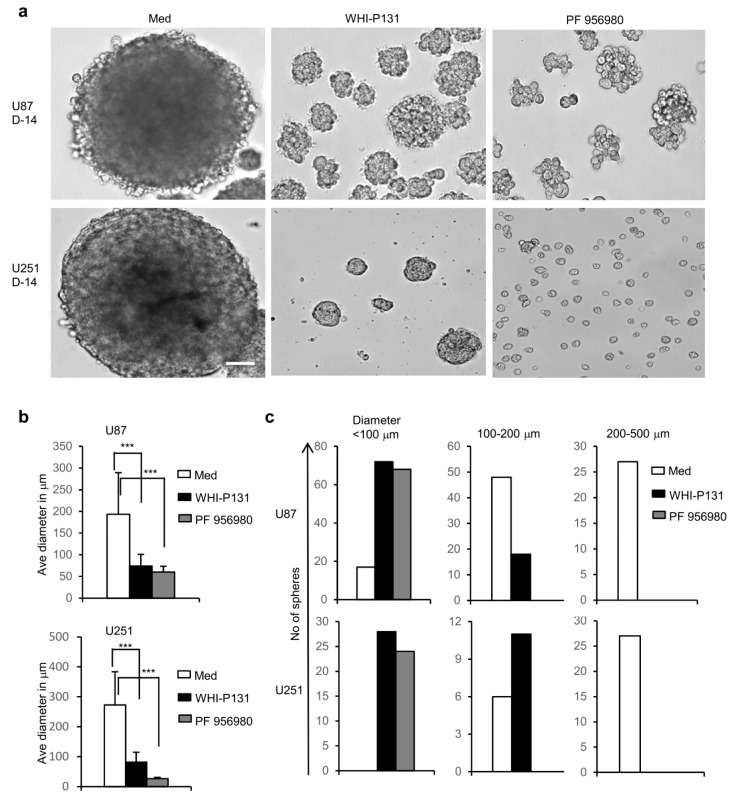
Effects of JAK3 inhibitors on neurosphere-forming ability of GBM cells. (**a**) Neurosphere formation in absence or presence of JAK3 inhibitors WHI-P131 (50 μM) or PF-956980 (250 μM) in U87 and U251 cultures 14 days after treatment. Scale bar: 100 μm. (**b**) Average size of neurospheres in U87 or U251 cells left untreated or treated with JAK3 inhibitors for 14 days in proliferation medium (*** *p* < 0.0001; unpaired t-test). (**c**) Distribution of neurospheres according to their size in U87 or U251 cultures in presence or absence of JAK3 inhibitors at day 14. Data are representative of five independent experiments.

Spheroid formation is the fundamental property of neural stem cells, and when cultured in a proliferation medium, they rapidly form neurospheres. This is also a parameter to assess their proliferative capacity. To analyze whether the JAK3 inhibitors WHI-P131 and PF-956980 could affect the stemness of GBM cells and their ability to form spheres, we treated U87 and U251 cells with the inhibitors and cultured them in a proliferation medium devoid of serum but containing epidermal growth factor (EGF) and basic fibroblast growth factor (FGF). Observation of the U87 cells at D-14 of culture revealed the formation of huge spheres in the untreated wells (with an average size of 200 μm), and this ability was strongly reduced in the WHI-P131- and PF-956980-treated cells (with an average size of 50 μm) (Figure 4a,b). In the JAK3-inhibitor-treated cells, there were spheres of very small sizes (<100 μm), and they did not grow over time, whereas the majority of spheres in the control cells were within the range of 100–500 μm (Figure 4c). Similarly, the untreated U251 cells formed huge spheres (with an average size of 300 μm), and the inhibitor-treated cells were very poor at forming spheres (with an average size of 50 μm) (Figure 4a,b). The WHI-P131-treated U251 cells formed smaller spheres, and the PF-956980-treated U251 cells failed to form spheres at all (Figure 4c). This trend of a strongly reduced ability to form neurospheres following JAK3 inhibition was already evident at D-7 (Supplementary Figure S3), after which the spheres in the medium-only wells further grew in size, whereas those in the JAK3-inhibitor-treated wells did not. Thus, both JAK3 inhibitors were very effective in negatively regulating the stemness of the GBM cells and strongly reduced their proliferation and sphere formation ability, which is in line with the strongly reduced proliferation we observed (Figure 1b,c).

### 3.5. Enhanced DNMT Activity in JAK3-Inhibitor-Treated GBM Cells

**Figure 5:**
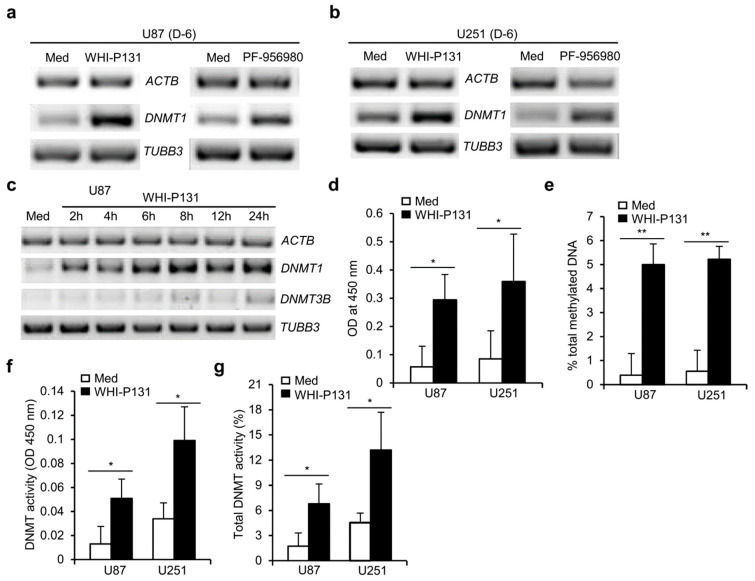
WHI-P131 and PF-956980 induce epigenetic changes in GBM cells. (**a**) Levels of *DNMT1* and *TUBB3* expression in WHI-P131- (50 μM) or PF-956980- (250 μM) treated U87 cells six days after culture, as revealed with semi-quantitative RT-PCR. (**b**) *DNMT1* and *TUBB3* expression in U251 cells six days after culture in presence or absence of WHI-P131 (50 μM) or PF-956980 (250 μM) treatment. (**c**) Levels of *DNMT1*, *DNMT3B*, and *TUBB3* expression in WHI-P131- (50 μM) treated U87 cells at indicated time points of culture, as revealed with semi-quantitative RT-PCR. In (**a**–**c**), *ACTB* expression was used as loading control. (**d**) OD 450 nm values for untreated and WHI-P131- (50 μM) treated U87 and U251 cells representing methylated DNA after 24 h drug treatment, as estimated with the MethylFlash™ Methylated DNA Quantification Kit (Epigentek) (U87: * *p* = 0.0185; U251: * *p* = 0.0212; paired t-test). (**e**) Estimation of % total methylated DNA in U87 and U251 cells 24 h after WHI-P131 (50 μM) treatment (U87: ** *p* = 0.0035; U251: * *p* = 0.0065; paired t-test). (**f**) Total DNMT activity represented as OD 450 nm values for U87 and U251 cells either treated or left untreated for 24 h with WHI-P131 (50 μM), as estimated using the EpiQuik™ DNA Methyltransferase Activity/Inhibition Assay Ultra Kit (U87: * *p* = 0.0467; U251: * *p* = 0.0392; paired t-test). (**g**) Estimation of % total DNMT activity in U87 and U251 cells 24 h after WHI-P131 (50 μM) treatment (U87: * *p* = 0.0272; U251: * *p* = 0.0330; paired t-test). Data are representative of three independent experiments and in (**d**–**g**) are presented as means ± sd.

To investigate the rapid effect of the JAK3 inhibitors on the inhibition of proliferation and induction of differentiation, we assumed these inhibitors most likely affect epigenetic changes in GBM cells. DNA methylation is an essential component of the epigenetic apparatus, which has been implicated in various types of cancer. DNA methyl transferases (DNMTs) are enzymes that methylate cytosine residues by transferring a methyl group from the universal donor S-adenosyl-L-methionine (SAM) to the 5-position of cytosine residues in DNA and thereby regulate their epigenetic status and gene regulation. There are four DNMTs (DNMT1, DNMT3A, DNMT3B, and DNMT3L) involved in mammalian development. Interestingly, both WHI-P131 and PF-956980 treatment of U87 and U251 cells showed increased expression of *DNMT1* (Figure 5a,b) compared with untreated cells, suggesting that the JAK3 inhibitors are able to alter the epigenetic state of GBM cells. The increase in *DNMT1* expression was quite rapid as it was already evident 2 h after inhibitor treatment (Figure 5c). An increase in *DNMT3B* was also observed in U87 cells, although it was less prominent than that in *DNMT1* (Figure 5c). Interestingly, the upregulation of *DNMT1* expression was specific to JAK3 inhibition as the expression of *TUBB3*, a pan-neuronal cell-specific marker, was unaffected at all time points analyzed (Figure 5a–c).

To check whether the increased *DNMT* expression was reflected in an increase in DNA methylation, we performed a DNA methylation analysis. As shown in Figure 5d, there was a significant increase in DNA methylation after 24 h of WHI-P131 treatment in both U87 and U251 cells compared with untreated control cells. The quantification of the methylated DNA following JAK3 inhibitor treatment revealed a ten-fold or more increase in methylated DNA in the treated U87 and U251 cells compared with untreated GBM cells (Figure 5e). As a confirmation of the increase in *DNMT* expression and enhanced levels of methylated DNA, we observed a significant increase in total DNMT activity in both the GBM cell lines 24 h after WHI-P131 treatment (Figure 5f). The quantification of the DNMT activity revealed a more than two-fold increase in the enzyme activity in both GBM cell lines following JAK3 inhibition (Figure 5g). These observations suggest that the inhibition of JAK3 activity could potentially alter the epigenetic status of GBM cells and thereby not only prevent their ability to proliferate but also induce their quick differentiation into mature stages.

### 3.6. JAK3 Inhibition Changes the Stemness of GBM Cells

**Figure 6:**
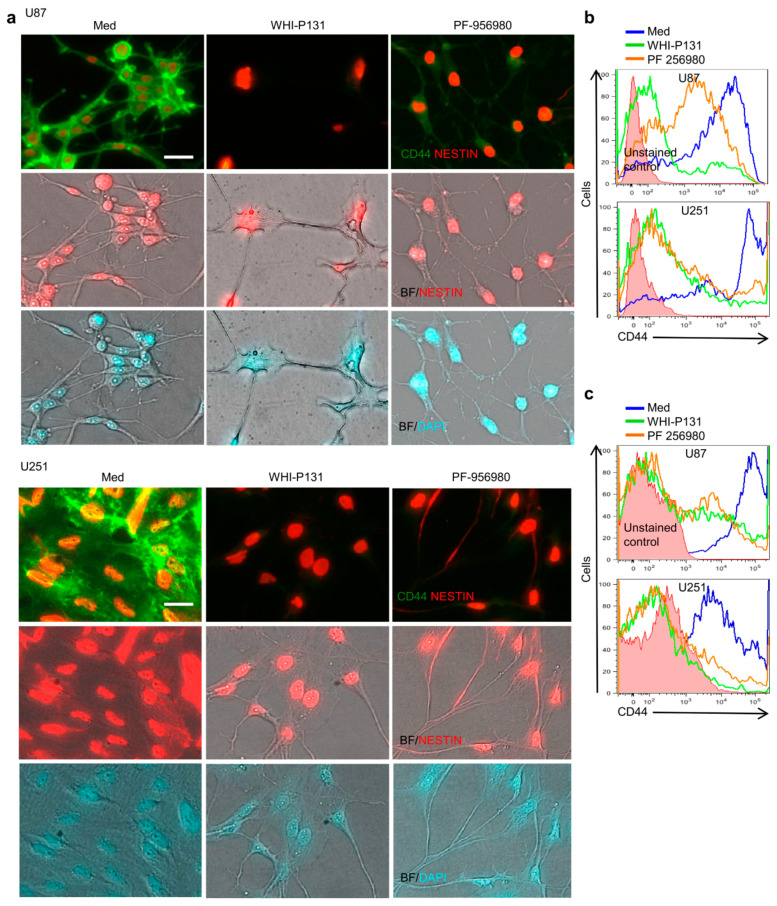
JAK3 inhibition suppresses the stemness of GBM cells. (**a**) Expression levels of GBM stem cell markers CD44 and NESTIN in presence or absence of WHI-P131 (50 μM) or PF-956980 (250 μM) in U87 and U251 cells six days after culture. DAPI was used for nuclear staining. Scale bar: 100 μm. (**b**) CD44 expression in untreated or WHI-P131- (50 μM) or PF-956980- (250 μM) treated U87 and U251 cells six days after culture in differentiation medium, as revealed with flow cytometry. (**c**) Flow cytometry analysis of CD44 expression in neurospheres from untreated or WHI-P131- (50 μM) or PF-956980- (250 μM) treated U87 and U251 cells ten days after culture in proliferation medium. Data are representative of four independent experiments.

As GBM cells are developmentally immature and retain neural stem cell features, they express several stem cell markers such as SOX2, CD44, NESTIN, PROMININ, etc., which critically regulate their tumorigenicity [25]. As the JAK3 inhibitors WHI-P131 and PF-956980 rapidly shut down GBM cell proliferation and neurosphere formation and induced differentiation into mature neuronal cells, we assumed that most likely, these inhibitors strongly interfere with the stemness of these cells and facilitate their maturation. We analyzed the changes in the expression of several neural-stem-cell-specific markers following JAK3 inhibitor treatment and interestingly observed a strong decrease in the expression of CD44 in both U87 and U251 after 6 days of culture (Figure 6a). CD44 has been implicated in GBM pathogenesis by maintaining stem-cell-like properties, and inhibition of its activities has been reported to reduce the severity of GBM [26,27]. Therefore, a strong downregulation of CD44 in JAK3-inhibitor-treated GBM cells is a promising observation. The inhibitor-treated cells appeared fully differentiated and lacked proliferation in contrast with the untreated control cells. However, under the same condition of JAK3 inhibition, NESTIN, another neural stem cell marker prominently expressed in GBM cells, was not affected and remained nuclear (Figure 6a). Also, the expression of other neural stem cell markers such as PROMININ and SOX2 was not affected by drug treatment (data not shown). This was true for both WHI-P131 and PF-956980 treatment of U87 and U251 cells after 6 days of culture. The downregulation of CD44 expression in U87 and U251 cells upon JAK3 inhibition both in the serum-containing differentiation medium and the EGF- and FGF-containing proliferation medium was further confirmed in flow cytometry experiments following 6 days and 10 days of drug treatment, respectively (Figure 6b,c). The changes in the expression of specific neural stem cell markers rather than a global shutdown of gene expression suggest that the JAK3 inhibitors act specifically and selectively. Therefore, they have potential use in therapy against GBM.

## 4. Discussion

GBM is a deadly tumor with a huge unmet clinical need. The therapeutic strategies available at present are grossly inadequate to have any impact on prolonging the survival of GBM patients. Although research so far has implicated several molecules to be the causative agents for GBM pathogenesis, a holistic picture to understand the pathogenesis and to apply a curative therapy to prolong the lifespan of patients is still very far away. The vigorous proliferation rate of these cells is a challenge, and several reagents have been tested to block this proliferation and kill the tumor cells. However, considering the genetic and mechanistic heterogeneity of GBM pathogenesis, a complete understanding of the growth and differentiation of GBM cells is essential to devise an efficient therapy to eliminate them.

Overactivity of JAK family kinases has been reported to play a role in various types of cancer, mostly hematological ones [28,29,30]. But recently, many groups have explored their involvement in GBM pathogenesis and by using small-molecule inhibitors of JAKs or downstream TF STAT3, have observed beneficial effects in in vitro and in vivo studies [20,31,32,33]. The JAK family member that has been extensively investigated in GBM is JAK2. However, so far, nothing has progressed to a successful clinical trial without having severe side effects or toxicity. JAK3’s involvement in GBM pathogenesis has been relatively less explored. We showed that by treating U87 and U251 GBM cells with two specific JAK3 inhibitors, WHI-P131 and PF-956980, JAK3 activity could be significantly downregulated, and this has tremendous beneficial effects in preventing tumor cell proliferation and inducing cellular differentiation (Supplementary Figure S4).

WHI-P131 and a related compound WHI-P154 have been previously reported to prevent GBM cell adhesion and migration [34]. WHI-P154 alone and a conjugate of EGF receptor-WHI-P154 have also been reported to induce cytotoxicity in GBM cells [35]. However, we did not observe any defect in cell adhesion or the induction of cytotoxicity at a range of WHI-P131 concentrations of up to 200 μM. We also tested WHI-P154 against both U87 and U251 cells and obtained similar results to those for WHI-P131 in terms of the inhibition of proliferation and induction of differentiation. PF-956980 has not been tested against GBM, but herein, we showed that it has a similar effect to that of WHI-P131 in preventing GBM pathogenesis, albeit at a higher concentration. However, even at these concentrations, they are not toxic either to the proliferating tumor cells or to the differentiated tumor cells, as we did not observe significant cell death at either early or late time points of the drug treatments. Thus, both inhibitors hold promise to be used against GBM to provide benefit to patients.

The presence of serum in culture media has been reported to induce the differentiation of neural stem cells into mature stages. However, despite the high serum amount in the culture medium and lack of neural growth factors such as EGF and FGF, these cells proliferated extensively without differentiation, showing the robustness of the tumorigenic phenotype of these cells. Both in the serum-containing differentiation medium and the EGF- and FGF-containing proliferation medium, U87 and U251 cells proliferated extensively. However, our findings show that a single treatment with either WHI-P131 or PF-956980 could not only effectively block their proliferation but also induced their differentiation into various neural lineage cells. The longest period we monitored was 75 days after a single dose of drug treatment, during which the cells remained alive and in a differentiated state. But we are sure they can be maintained in this non-tumorigenic differentiated condition as long as they are maintained with the drugs. This is an observation of huge interest as it suggests either these drugs or a more refined form of these drugs could potentially be beneficial in GBM treatment with fewer side effects.

The current treatment procedure of surgical resection followed by temozolomide therapy does not prevent tumors from developing resistance and leads to the relapse of more vigorous tumors. There are several limitations to the current treatment strategy. First of all, it is almost impossible to precisely remove all of the tumor tissue with surgery. The fact that the remaining tumor cells rapidly proliferate and re-form the tumor mass is the main roadblock to successful therapy. Our findings suggest that following the surgical removal of most of the tumor tissue, the remaining tumor cells, irrespective of whether they are cancer stem cells (CSCs) or non-stem tumor cells, could be kept in a non-proliferative, non-tumorigenic differentiated state, which might help to prolong the lifespans of GBM patients. We have shown that both WHI-P131 and PF-956980 treatment effectively act on the U87 and U251 cell populations, including CSCs, and they also downregulate the stemness-related genes.

Our observations regarding epigenetic modifications, such as increased DNMT activity, might be an important factor following JAK3 inhibition in preventing GBM cell proliferation and inducing differentiation. Methylation at the O^6^-methylguanine-DNA methyltransferase (MGMT) gene promoter has been detected in nearly 50% of high-grade gliomas and is being used as a good prognostic marker for effective chemotherapy. Based on our results, we can assume an enhanced *MGMT* methylation in JAK3-inhibitor-treated U87 and U251 cells. Whether this contributes to the observed effects of the JAK3 inhibitors in our study needs further analysis. However, this raises the interesting possibility of treating MGMT unmethylated cases of GBM with JAK3 inhibitors to make them more susceptible to chemotherapy using alkylating agents. This will potentially help many GBM patients prolong their lifespans.

In the current study, we did not look into other epigenetic changes that could be involved in the phenotype we observed following JAK3 inhibition. However, how these epigenetic changes occur, the kinetics, and the molecules involved in this process in GBM cells need further investigation. That the GBM cells remain in a differentiated state for a long time even with only a single treatment of the inhibitors holds promise for future refinement to develop more effective forms of these drugs working at a lower concentration to prevent GBM progression.

## 5. Conclusions

Our results show that the inhibition of JAK3 activity has multiple potential benefits in controlling GBM cell proliferation and relapse. Detailed molecular mechanisms of how these inhibitors suppress cell proliferation and induce differentiation need to be elucidated to make them therapeutically relevant. One possibility could be that these JAK3 inhibitors alter the epigenetic status of GBM cells, as we have shown in the case of increased DNMT activity following WHI-P131 and PF-956980 treatment. Most likely, enhanced DNMT activity results in the methylation of tumor-promoting genes and suppresses their activity. This needs to be explored and if true, can be manipulated to control GBM pathogenesis and relapse following JAK3 inhibition.

## Data Availability

Not applicable.

## Supplementary Materials

The following supporting information can be downloaded at: https://www.mdpi.com/article/10.3390/cells12212547/s1, Figure S1: WHI-P131 and PF-956980 treatment do not induce cell death in GBM cells; Figure S2: WHI-P131 and PF-956980 induces differentiation of GBM cells to neuronal lineage cells; Figure S3: Effects of JAK3 inhibitors on neurosphere forming ability of GBM cells; Figure S4: Model showing the effects of JAK3 activity and its inhibition by JAK3 inhibitors on GBM cell fate.
