# Supplementary material for: JAK3 Inhibition Regulates Stemness and Thereby Controls Glioblastoma Pathogenesis
Source: Cells. 2023 Oct 30;12(21):2547. doi: 10.3390/cells12212547 (PMC10649349; doi:10.3390/cells12212547)
Supplement: Supplementary file 1 [file cells-12-02547-s001.zip › cells-2693442-supplementary.pdf]

Supplementary Figure S1

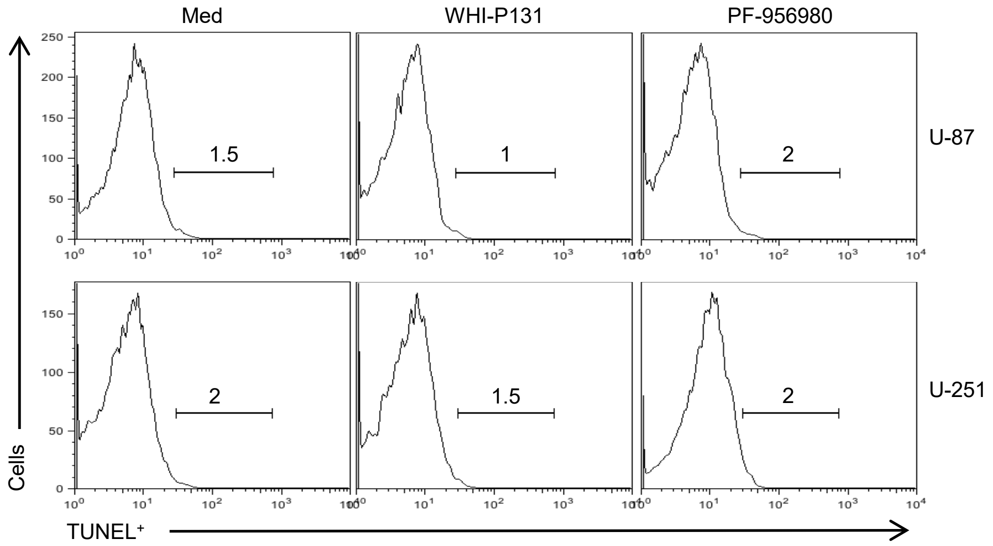

Figure S1. **WHI-P131 and PF-956980 treatment do not induce cell death in GBM cells.** TUNEL assay showing the distribution of live (TUNEL$^-$) and dead (TUNEL$^+$) cells evaluated by flow cytometry in 48h culture of U87 and U251 cells with or without treatment of JAK3 inhibitors WHI-P131 (50µM) or PF-956980 (250µM). Data are representative of two independent experiments.

Supplementary Figure S2

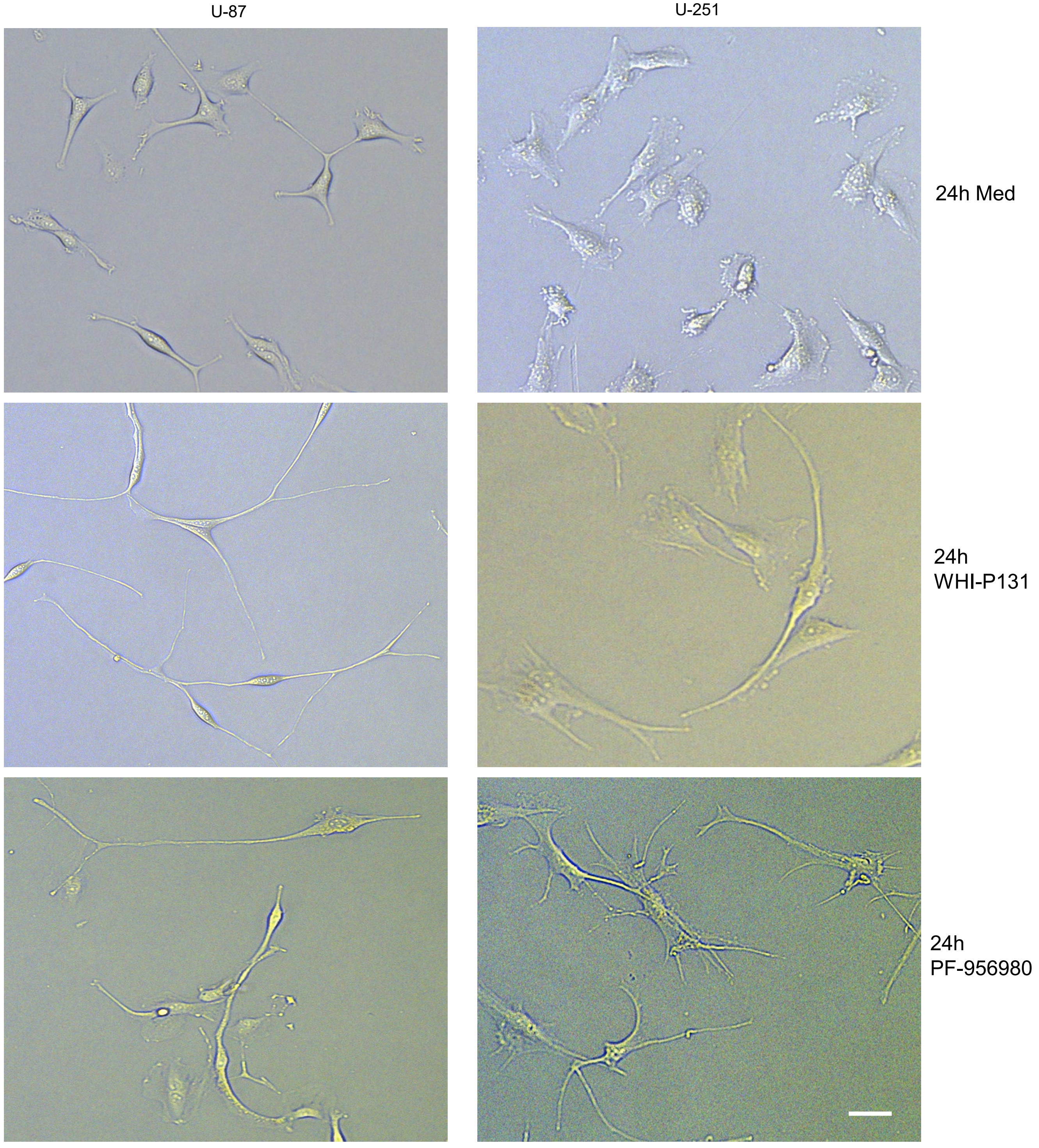

Figure S2. **WHI-P131 and PF-956980 induces differentiation of GBM cells to neuronal lineage cells.** Status of U87 and U251 cells proliferation or differentiation at 24h of culture with or without treatment of JAK3 inhibitors WHI-P131 (50μM) or PF-956980 (250μM). Data are representative of ten independent experiments. Scale bar 75 μm.

Supplementary Figure S3

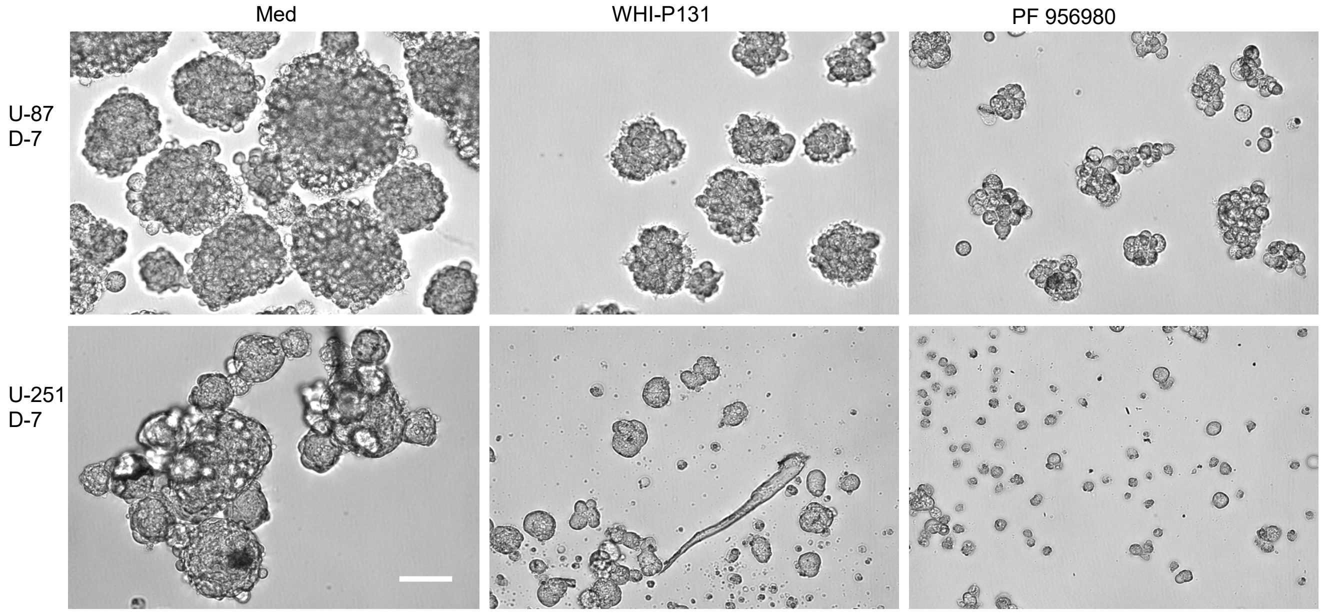

Figure S3. **Effects of JAK3 inhibitors on neurosphere forming ability of GBM cells.** Neurosphere formation in absence or in presence of JAK3 inhibitors WHI-P131 (50μM) or PF-956980 (250μM) in U87 and U251 cultures 7 days after treatment. It is clearly evident that JAK3 inhibitors strongly reduced the sphere size and thereby the ability of U87 and U251 cells to proliferate. Scale bar 100 μm.

Supplementary Figure S4

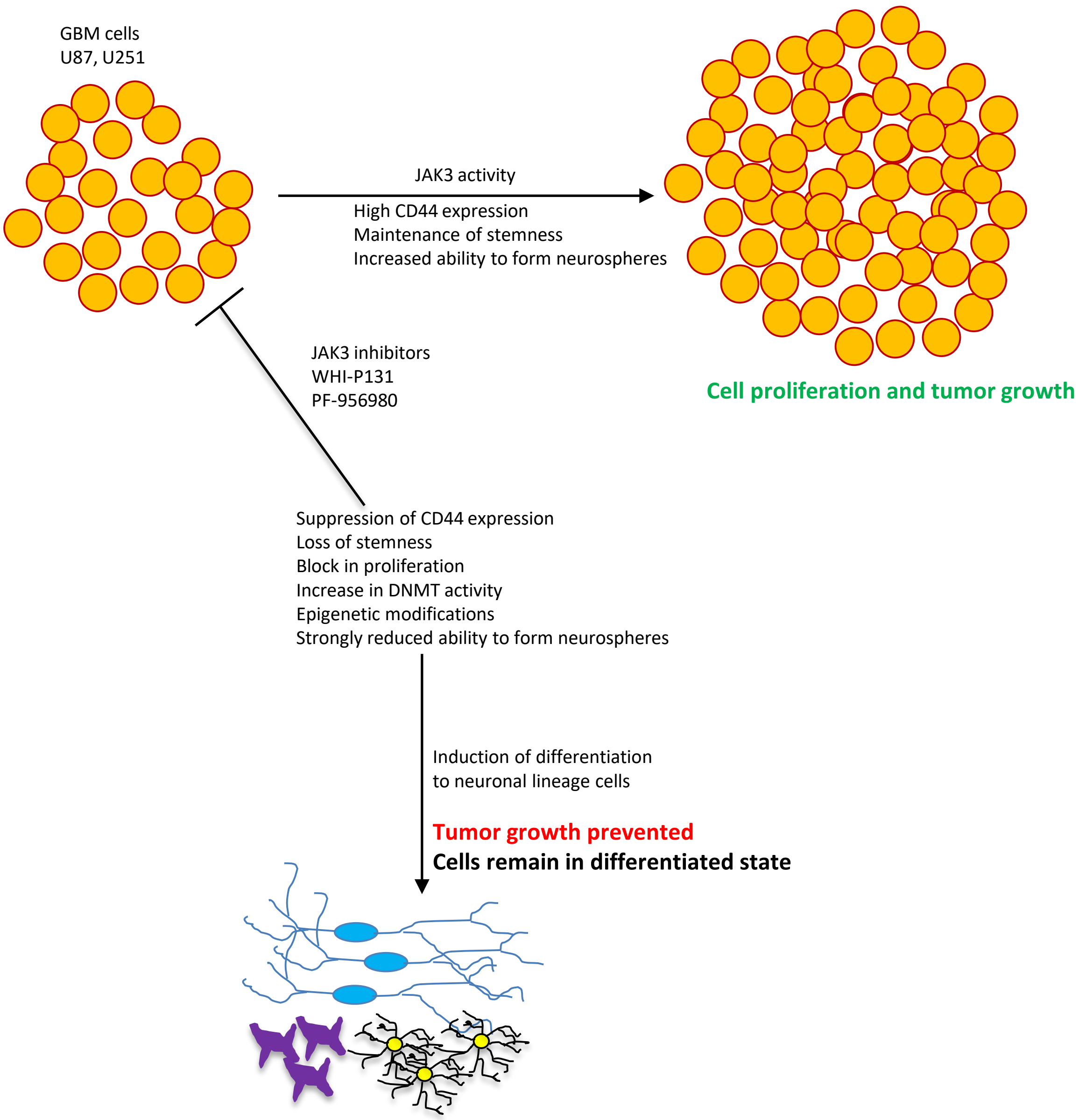

Figure S4. Model showing the effects of JAK3 activity and its inhibition by JAK3 inhibitors on GBM cell fate. With uninhibited JAK3 activity both U87 and U251 cells proliferate extensively and the tumor grows. Treatment of JAK3 inhibitors WHI-P131 (50μM) or PF-956980 (250μM) effectively blocks proliferation, induce epigenetic changes leading to differentiation into neuronal lineage cells. As a result, tumor growth is prevented and the cells remain in differentiated state.
